# Precision Medicine in Hematology 2021: Definitions, Tools, Perspectives, and Open Questions

**DOI:** 10.1097/HS9.0000000000000536

**Published:** 2021-02-17

**Authors:** Peter Valent, Alberto Orfao, Stefan Kubicek, Philipp Staber, Torsten Haferlach, Michael Deininger, Karoline Kollmann, Thomas Lion, Irene Virgolini, Georg Winter, Oliver Hantschel, Lukas Kenner, Johannes Zuber, Florian Grebien, Richard Moriggl, Gregor Hoermann, Olivier Hermine, Michael Andreeff, Christoph Bock, Tariq Mughal, Stefan N. Constantinescu, Robert Kralovics, Veronika Sexl, Radek Skoda, Giulio Superti-Furga, Ulrich Jäger

**Affiliations:** 1 Department of Medicine I, Division of Hematology & Hemostaseology, Medical University of Vienna, Austria; 2 Ludwig Boltzmann Institute for Hematology and Oncology, Medical University of Vienna, Austria; 3 Servicio Central de Citometria, Centro de Investigacion del Cancer (IBMCC; CSIC/USAL), IBSAL, CIBERONC and Department of Medicine, University of Salamanca, Spain; 4 CeMM Research Center for Molecular Medicine of the Austrian Academy of Sciences, Vienna, Austria; 5 MLL Munich Leukemia Laboratory, Munich, Germany; 6 Division of Hematology and Hematologic Malignancies, University of Utah, Huntsman Cancer Institute, Salt Lake City, Utah, USA; 7 Institute of Pharmacology and Toxicology, University of Veterinary Medicine Vienna, Austria; 8 Children’s Cancer Research Institute, Vienna, Austria; 9 Department of Nuclear Medicine, Medical University of Innsbruck, Austria; 10 Institute of Physiological Chemistry, Faculty of Medicine, Philipps-University of Marburg, Germany; 11 Pathology of Laboratory Animals, University of Veterinary Medicine, Vienna, Austria; 12 Research Institute of Molecular Pathology (IMP), Vienna, Austria; 13 Institute for Medical Biochemistry, University of Veterinary Medicine Vienna, Austria; 14 Institute of Animal Breeding and Genetics, Unit for Functional Cancer Genomics, University of Veterinary Medicine Vienna, Austria; 15 Imagine Institute Université Paris Descartes, Sorbonne, Paris Cité, Paris, France; 16 Department of Hematology, Necker Hospital, Paris, France; 17 University of Texas MD Anderson Cancer Center, Houston, Texas, USA; 18 Division of Hematology & Oncology, Tufts University Medical Center, Boston, Massachusetts, USA; 19 de Duve Institute and Ludwig Cancer Research Brussels, Université catholique de Louvain, Brussels, Belgium; 20 Departement of Biomedicine, University of Basel, Switzerland.

## Abstract

During the past few years, our understanding of molecular mechanisms and cellular interactions relevant to malignant blood cell disorders has improved substantially. New insights include a detailed knowledge about disease-initiating exogenous factors, endogenous (genetic, somatic, epigenetic) elicitors or facilitators of disease evolution, and drug actions and interactions that underlie efficacy and adverse event profiles in defined cohorts of patients. As a result, precision medicine and personalized medicine are rapidly growing new disciplines that support the clinician in making the correct diagnosis, in predicting outcomes, and in optimally selecting patients for interventional therapies. In addition, precision medicine tools are greatly facilitating the development of new drugs, therapeutic approaches, and new multiparametric prognostic scoring models. However, although the emerging roles of precision medicine and personalized medicine in hematology and oncology are clearly visible, several questions remain. For example, it remains unknown how precision medicine tools can be implemented in healthcare systems and whether all possible approaches are also affordable. In addition, there is a need to define terminologies and to relate these to specific and context-related tools and strategies in basic and applied science. To discuss these issues, a working conference was organized in September 2019. The outcomes of this conference are summarized herein and include a proposal for definitions, terminologies, and applications of precision and personalized medicine concepts and tools in hematologic neoplasms. We also provide proposals aimed at reducing costs, thereby making these applications affordable in daily practice.

## Introduction

The terms “precision medicine” and “personalized medicine” are widely used in public media, healthcare systems, and in the scientific community. These terms, and other related terms, such as “P4 medicine,” “stratified medicine,” “genomic medicine,” or “evidence-based medicine,” are often used interchangeably to describe disease-specific and patient-related approaches that are based on our improved knowledge of the clinical impact of large-scale genetic, molecular, epigenetic, metabolic, and functional profiles on the individual patient’s diagnosis, prognosis, and outcome.^1-5^

Collectively, none of these terms are defined precisely, and people often mean different things when using these terms and are not aware of the definitions and interpretations that are employed by physicians and scientists in various contexts.^1-5^ This is probably due to the many disciplines (research, laboratory-based, clinical) and stakeholders involved and the lack of generally accepted definitions and terminologies in this young field of medicine. Especially the terms “precision medicine” and “personalized medicine” have been used interchangeably, although most experts believe that these terms may have different meanings or at least indicate nuances in the context of application and interpretation.^1-5^ There have also been general concerns regarding the applicability and appropriate use of these terms in recent years.^4-7^

Hematology is a well-developed discipline where the concepts of precision medicine and personalized medicine have, in part, already been implemented successfully.^8-11^ A highlighting example is the translation of BCR-ABL1-targeting concepts from preclinical development stages into applied hematology in chronic myeloid leukemia (CML) using specific drugs like imatinib.^12-14^ Major research efforts and the resulting insights into disease evolution have led to the development of second and third generation BCR-ABL1 tyrosine kinase inhibitors (TKI) in this disease, and precise knowledge about drug interaction profiles and related side effects that the individual TKI may provoke, have further advanced the field.^15-20^ As a result, BCR-ABL1 inhibitors are now used with great precision in various patient cohorts, based on disease-specific molecular and patient-related variables (age, comorbidities, risk of developing side effects) following the principles of personalized medicine.^21-26^

However, even in applied hematology, questions remain. For example, it remains open how precision medicine tools can successfully be implemented in healthcare systems in various countries and whether all diagnostic approaches, prognostic tools, and therapies are affordable. Moreover, diagnostic tools and emerging drugs are not always validated vigorously in all contexts and relevant patient cohorts. Furthermore, clinical trials are not always designed and resourced to evaluate all precision medicine aspects. In addition, there is an emerging need to establish terminologies and to provide precise definitions in the context of hematologic neoplasms.

To discuss these points, a working conference, including experts in clinical, translational, and basic hematology from various countries, was organized in Vienna in September 2019. The outcomes of this conference are summarized herein and include a proposal for definitions and terminologies, recommendations for the application of precision medicine tools in hematologic neoplasms, and proposals aimed at reducing costs with the hope of making precision medicine and personalized medicine approaches affordable and thus broadly applicable in the foreseeable future.

## Historical aspects and principles

The basic principles of personalized medicine, namely to “apply most appropriate medicines for the right patient at the right time,” are quite old. A systematic description of what we could call a theory of personalized medicine today was already developed by the ancient Greeks around Hippocrates (460-370 BC).^27-29^ In fact, Hippocrates himself emphasized the importance of individualizing the patient’s management, claiming that “it is often more important to know what person has a disease than to know what specific type of disease it is.”^27-29^ This example already points at the subtle difference between precision medicine and personalized medicine. However, at the time of Hippocrates, precise knowledge about the etiology and mechanisms of disease was lacking. Robust precision medicine tools were developed much later when the principles of chemistry, microbiology, and cell biology had been developed in the second part of the 19th century. At that time, Paul Ehrlich (1854-1915) and others working in the “new” fields of chemistry, microbiology, and pharmacology were the first to define the principles of precision medicine.^30-34^ However, even they had to face the fact that precision medicine tools alone are not able to overcome all obstacles and hurdles in medicine, as patient-related factors and side effect profiles represent clear limitations regarding the intention to offer the best (most effective) therapy to all affected individuals.^30-35^ In other words, it soon turned out that precision medicine concepts have to be extended and complemented by a personalized approach in order to manage all subgroups of patients in an optimal (personalized) manner.

During the past 3 decades, the rapidly expanding fields of genome medicine and digital medicine, as well as the many new tools and technologies created to work “fast and large-scale” in these fields, have substantially pushed forward precision medicine and personalized medicine concepts in various disciplines.^1-7,36-39^ Together with the development of new anticancer therapies and new concepts about the evolution and progression of malignant disorders, these emerging tools and technologies have revolutionized clinical hematology and oncology in recent years, and have improved diagnostics, prognostication, and therapy substantially.^40-44^

However, as mentioned, there is still an ongoing debate concerning the correct application and use of the terms “precision medicine” and “personalized medicine.” In the following paragraphs, we will first discuss opinions and views concerning the appropriate use of the terms “precision medicine” and “personalized medicine” (1) in general and (2) specifically in hematologic neoplasms. Then, we will provide a proposal for these definitions and terminologies in hematology contexts.

## Previous proposals and dispute about application and applicability

In the past few decades, we have witnessed an increasing number of academic disputes and policy discourses around the terms “precision medicine,” “stratified medicine,” and “personalized medicine.”^1-7,36-39^ Unfortunately, these terms are increasingly used in unrelated contexts by public media, healthcare providers, funding sources, politicians, and other stakeholders.^1-7^ As a consequence, the application and usefulness of these terms were questioned. For example, Pokorska-Bocci et al^3^ reported in 2014 that personalized medicine is an umbrella term covering many concepts whose meanings have become looser and interchangeable over time. Others have pointed at the fact that criteria and definitions for the terms “precision medicine” and “personalized medicine” have been adjusted variably by stakeholders, depending on their expectations and aims.^4-7^ In 2015, the European Commission (Council of the European Union) proposed that the term “personalized medicine” should refer to a medical concept or model involving the individual person’s phenotypes and genotypes for tailoring the right therapeutic strategy for the right person at the right time (Hippocrates’s wording) and/or to determine predisposing factors with the aim to deliver specific and timely prevention.^45^ This definition was also employed by the Horizon 2020 program of the European Commission in 2017.^46,47^ Their advisory group recommended the term “personalized medicine” over other terms by noting that this term would best reflect the primary goal of the Horizon 2020 program, which was to effectively tailor prophylactic approaches and treatments, based on an individual’s “personal profile.”^48^ However, optimal treatment individualization is difficult to achieve with current facilities and tools in most areas of applied medicine.^1,49^

All in all, there is an obvious need to propose definitions, terminologies, and applications in the emerging fields of precision medicine and personalized medicine. In the following sections, we propose such definitions for hematologic neoplasms.

## Proposed basic definitions for precision medicine and personalized medicine in the context of hematologic neoplasms

Research in hematologic malignancies is often considered to be slightly ahead of time compared with other disciplines, both in basic science and in translational contexts. Therefore, it is not surprising that in contrast to other disease areas, the basics around “precision medicine” and “personalized medicine” are more established and readily applicable in hematology. In other words, it is particularly realistic to discuss the scope, application, and definition of the terms “precision medicine” and “personalized medicine” in the context of hematology. Our faculty is of the opinion that these 2 terms are highly relevant and that the related concepts are already applied in the field of hematologic malignancies. Although both terms allude to disease-related and patient-related aspects in general, precision medicine appears to be a more basic term. Whereas precision medicine is more closely related to disease-specific features and molecular or cellular interactions and explores how these features impact the patient’s diagnosis, prognosis, and outcome in general, personalized medicine also (in addition) relates to multiple aspects of the individual patient in a comprehensive way, including (among others) age, gender, genetic features, epigenetic variables, and comorbidities. Sometimes, personalized medicine even includes mental, social, and/or psychological aspects. With regard to drug development and drug application, precision medicine is focusing more on drug-target interactions and drug efficacy in cells and patients, whereas personalized medicine has to take into account multiple or even all patient-related aspects, including age, gender, pharmacologic variables, drug-relevant metabolic variables, adverse event profiles, and comorbidities. With increased frequency, personalized medicine also has to consider drug-drug interactions. Finally, personalized medicine is considering multiple patient-related aspects in order to develop risk scores, disease-preventing strategies, or concepts for early intervention.

When a drug is developed for use in a patient population based on large data sets (derived from patients or other sources) and on specific interactions between the drug and known molecular targets and/or target cells (derived from patients or other sources), the approach can be labeled as “precision medicine-based,” but should not be regarded as a personalized medicine approach as it remains unknown what subset(s) of patients under what conditions would benefit from the drug, and what (and how many) subset(s) of patients may even suffer from the consequences of the approach.

The definitions we propose for “precision medicine” and “personalized medicine” in hematologic neoplasms (basic and applied hematology setting) are shown in Table 1.

**Table 1:** Proposed Definitions of Precision Medicine, Personalized Medicine, and EBM in the Context of Hematologic Neoplasms.

**Precision medicine**
• Concept or approach in preclinical, translational, or applied medicine that takes 1 or more defined molecules, cells, and/or interactions between (networks of) molecules and cells into account with the aim to improve diagnostics, prognostication, prevention, and/or therapy in hematologic neoplasms
**Personalized medicine**
• Concept or approach in translational or applied medicine that takes 1 or more clinically relevant molecules, cells, and/or interactions between (networks of) molecules and cells as well as several (or all) relevant patient-related factors into account with the aim to apply the optimal diagnostic procedures, algorithms, and prognostication tool and to select the optimal preventive strategy and/or treatment approach for the individual (the right) patient or patient cohort at the right time in hematologic neoplasms
**EBM^a^**
• Concept or approach in applied hematology that is based on generally accepted, published evidence^b^, with recognition of the evidence-source, evidence level, applicability, and the consequences and risk profiles in individual patients

^a^EBM is closely related to precision medicine and personalized medicine. However, EBM is a more general approach that can also be based on empiric studies demonstrating significant effects in certain patient cohorts without knowing a precise mechanism or target and without knowing why a specific subset of patients would or would not respond or benefit from the approach.

^b^Published evidence should mean that the data have been published in a peer-reviewed journal. Multiple confirming publications can further increase the overall impact and general acceptance of the approach.

EBM = evidence-based medicine.

Precision medicine is a concept or approach in preclinical, translational, or applied medicine that takes 1 or more defined molecules, cells, and/or interactions between (networks of) molecules and cells into account, with the aim of improving diagnostics, prognostication, prevention, and/or treatment of hematologic neoplasms.

Personalized medicine is a concept or approach in translational or applied medicine that takes 1 or more clinically relevant molecules, cells, and/or interactions between (networks of) molecules and cells as well as several (or all) relevant patient-related factors into account, with the aim of applying optimal diagnostic procedures and optimal prognostication tool(s), and of selecting the optimal preventive strategy and/or treatment approach for the right (defined) patient (or patient cohort) at the right time in hematologic neoplasms (Table 1). The definition of “personalized medicine” is thus in line with the ancient principles defined by Hippocrates and the more recent definitions employed by the European Commission in the Horizon 2020 program. As such, it also represents an extension of the “precision medicine concept,” with a broader scope of application, based on individual patient-related variables.

Our faculty also discussed the relationship between personalized medicine and the frequently used term “evidence-based medicine” (EBM) coined in the early 1990s. Our faculty is of the opinion that the scope of EBM is closely related to the definitions of personalized medicine. However, we also believe that subtle differences exist. In fact, EBM is a more general approach that can also be based on empiric studies and data published in peer-reviewed journals demonstrating significant effects in certain patient cohorts without knowing the mechanism or target and without knowing why a specific subset of patients would or would not respond or benefit from the approach (Table 1).

Our faculty also discussed the primary goals and perspectives of precision and personalized medicine in hematologic neoplasms and application-specific “endpoints” that are the most important triggers for developing precision medicine tools and approaches. These goals and endpoints are shown in Table 2. In precision medicine, the goals focus on outcomes and endpoints in patient cohorts, independent of the individual patient’s overall situation. By contrast, in personalized medicine, the goal is to focus on the optimal strategy and outcome in the individual patient, depending on multiple disease-related and numerous patient-specific variables (Table 2).

**Table 2:** Goals and Endpoints in Precision Medicine and Personalized Medicine in Hematologic Neoplasms and Premalignant Stages.

**Goals and endpoints in precision medicine**
• Early recognition of premalignant stages of a malignancy (aggressive neoplasm)
• Prevention of development of a malignancy with optimal prophylactic approaches
• Establishing the correct diagnosis and variant of a neoplasm in all subsets of patients
• Establishing the prognostic profile of the patient by applying prognostic scores
• Establishing the treatment plan by utilizing molecular and cell-based information and biomarkers, including genetic, somatic, epigenetic, pathway-related, metabolic, and/or proteome-related patterns and interactions, including preinterventional in vitro drug testing
• Defining the optimal tools and parameters to measure treatment responses and to monitor long-term outcomes in patients
**Goals and endpoints in personalized medicine^a^**[Table-fn T2Fn1]
• Early recognition of the individual’s risk to carry a premalignant stages of a malignancy
• Age-, sex-, and comorbidity-adjusted selection of the optimal prophylactic approach to prevent the development of a malignancy in individual patients
• Defining the individual’s risk of malignant transformation in indolent hematologic neoplasms using prognostic scores
• Define optimal monitoring strategies in individual patients before, during, and after therapy
• Establishing the correct diagnosis and variant of a neoplasm as well as its etiology in individual patients by taking disease-related and patient-related factors into account^b^
• Establishing the prognosis of the individual patient concerning survival, disease-free survival, expected quality of life, and treatment-related death and morbidities (adverse events) by applying prognostic scores and by taking additional patient-related variables into account
• Selection of the optimal therapeutic approaches and optimal timing of therapy in individual patients, based on disease-related and patient-related variables, such as age, sex, genetic and ethnic background, comorbidities, social and geographic aspects, psychological and mental parameters, training status (fitness), cognitive functions, and others
• Defining the optimal way of measuring treatment responses and monitoring long-term outcomes in individual patients, depending on disease-related and patient-related variables

^a^Endpoints relate in part to the general definition of personalized medicine proposed by Schleidgen et al.^6^

^b^Patient-related factors would, eg, be mutagenic events (such as radiation), which would change the diagnosis of, eg, AML to secondary AML or therapy-related AML.

AML = acute myeloid leukemia.

All in all, personalized medicine is operating beyond the scope of precision medicine, as it also takes into account multiple patient-related variables, with the aim of selecting patient subsets and individual patients for “optimal” personalized (individualized) intervention by balancing between efficacy, side effects (potential), quality of life, and the patient’s expectations. As mentioned, personalized medicine also has to consider comorbidities and possible drug-drug interactions in each case.

## Precision medicine tools and role of basic research

A number of different tools have been developed in the context of precision medicine in basic, translational, and applied hematology. A complete review of all these tools and facilities is beyond the scope of this article. Pertinent examples are shown in Table 3. A big advantage in research in hematologic neoplasms is that the neoplastic cells can be obtained easily, particularly in leukemia patients. One focus in precision medicine in hematology is large-scale screening for new genetic and somatic lesions that are relevant in the context of hematologic neoplasms (Table 3).^50-58^ Several tools required to discover such new lesions and to relate them to functional properties of cells and related phenotypes in patients have been developed in the past 20 years. High-throughput sequencing technologies and the related bioinformatics approaches and tools are now readily available and are able to decipher not only single lesions and vulnerabilities but also complex aberration patterns and subclonal evolution pathways in neoplastic cell populations.^50-58^

**Table 3:** Tools and Approaches Employed in Precision Medicine in Hematologic Neoplasms.

**Diagnostic/prognostic (examples**)
• Molecular screens for mutation profiles/patterns (NGS panels) to support diagnosis, the variant of diseases, and the prognostic grade/subset in individual patients
• Measurement of MRD levels by precise imaging technologies (scans), flow cytometry, PCR, immunohistochemistry, or other methods
• Discovery and preclinical development of new specific diagnostic or prognostic markers (biomarkers) by exploring patient-derived, neoplastic cells or generally available big data sets prepared from patient-derived cells
• Genetic screens for mutations to support the diagnosis (hereditary diseases or predispositions) and prognostication (prognostic gene patterns)
• Antibody-based cell phenotyping of neoplastic cells to establish or support the diagnosis and to assist in prognostication in individual patients
• High-capacity chromosome analyses and multicolor FISH studies to support the diagnosis, the variant of diseases, and the prognostic grade/subset in individual patients
• Advanced omics approaches and tools, including genomics, transcriptomics, proteomics, metabolomics, pharmacogenomics, and multiomics in bulk samples and at single-cell level, with the aim to increase precision in diagnosis and prognostication
• Multidrug testing approaches predicting responses of patient-derived cells to single drugs or to drug combinations in individual patients
• Application of large-scale data sets and bioinformatics to define patient subsets and to confirm the diagnosis, the disease variant, or to support prognostication
• Advanced imaging systems combining bioimaging, tomography, and radiolabel scans to detect neoplastic cells in various organs and/or lymph node involvement
**Therapeutic (examples**)
• Discovery and preclinical development of therapeutic targets by exploring patient-derived, neoplastic cells, or big data sets prepared from patient-derived cells
• Application of specific targeted drugs (antibody-based, cell-based, small molecules, others) based on target expression and function in (on) neoplastic cells: (1) in phase II-IV clinical trials (to confirm efficacy) or (2) in practice (approved by health authorities)
• Growth factor therapy based on a confirmed deficiency or inappropriate production of cells or growth factors in a demand-situation (eg, injection of erythropoietin as comedication in patients with low endogenous erythropoietin in hematologic neoplasms)
• Combination of therapeutic approaches to eradicate the disease and disease-related stem cells to develop curative (disease-eradicating) therapies in defined patient cohorts

FISH = fluorescence in situ hybridization; MRD = minimal residual disease; NGS = next-generation sequencing; PCR = polymerase chain reaction.

Moreover, basic science tools have been applied to identify and develop clinically relevant biomarkers in recent years.^11,59-64^ In addition, drug development programs have been pushed forward by developing novel, advanced technologies, tools, and by using cell line models as well as primary, patient-derived cells (Table 3). While cell line models are often used to investigate specific targets, biomarkers, cell components, or target combinations, primary cells are the most relevant preclinical tool precision medicine should employ to develop reasonable therapeutic concepts using drugs or drug combinations. In fact, only the primary neoplastic cells contain all genetic, somatic, and biochemical (abnormal) properties that act together in a patient-specific manner to provide the malignant phenotype that has to be addressed in research, translational testing, and therapy. Therefore, precision medicine-related technologies are increasingly concentrating on primary neoplastic (stem) cells. One example and interesting approach is multidrug testing to predict responses of neoplastic cells to antineoplastic drugs and drug combinations.^65-71^

Over the past 30 years, a growing number of tools and facilities supporting the implementation of precision medicine concepts in hematology have been translated from basic science into clinical practice. These include, among others, computer-assisted (automated) microscopy, advanced multicolor flow cytometry technologies, fluorescence in situ hybridization, highly sensitive polymerase chain reaction technologies, next-generation sequencing, and multidrug testing facilities.

Additional diagnostic precision medicine-based tools and procedures successfully introduced in applied hematology include certain imaging techniques (like PET CT or radioisotope-based approaches), automated (robot-based) immunostaining facilities in hematopathology, or novel bioinformatics systems.

Several emerging therapeutic precision medicine concepts are based on our knowledge of target expression profiles of neoplastic (stem) cells, interactions between neoplastic cells and the supporting microenvironment (stem cell niche), the effects of various immune cells, and endogenous suppressor molecules on neoplastic cells. Related clinical applications include, among others, the development and use of (1) specific inhibitors of oncogenic kinases, (2) antibodies directed against critical surface molecules, (3) antibody-toxin or antibody-drug conjugates, (4) chimeric antigen receptor (CAR)-T or CAR-natural killer cells directed against neoplastic cells, (5) agents directed against immune checkpoint molecules, and (6) bispecific or trispecific antibodies capable of recruiting effector cells and/or effector molecules to destroy neoplastic cells (Table 4). In addition, new strategies employ more effective and less toxic drug combinations, including priming strategies and synthetic lethality concepts (Table 4). A detailed description of all these technologies and approaches is beyond the scope of this article. We refer the reader to the available literature.

**Table 4:** Specific New Therapeutic Concepts Developed and Translated in Precision Medicine in Hematologic Neoplasms between 2000 and 2020 (Examples).

Type of therapy	Translation stage
Oncoprotein-targeting kinase inhibitors	Approved and routinely applied in various myeloid and lymphoid neoplasms
Cell-specific antibody constructs to destroy neoplastic cells	Approved and routinely applied in various lymphoid neoplasms but only in a few myeloid neoplasms (eg, GO)
Bispecific antibodies	Applied in some lymphoid neoplasms but not in myeloid neoplasms (except in trials)
Immune checkpoint targeting antibodies	Applied routinely in several lymphoid neoplasms (eg, Hodgkin disease) and in clinical trials in myeloid neoplasms
Immune checkpoint regulating drugs	Preclinical development and clinical trials
CAR-T cell therapies	Approved in a few lymphoid neoplasms (eg, R/R ALL, R/R DLBCL) and tested in clinical trials in myeloid neoplasms
CAR-NK cell therapies	Preclinical studies and clinical trials
Novel degrader-type drugs	Preclinical experimental studies
New drug combination strategies such as priming concepts or synthetic lethality	Preclinical studies and clinical trials

ALL = acute lymphoblastic leukemia; CAR-T = chimeric antigen receptor T cell; DLBCL = diffuse large B-cell lymphoma; GO = gemtuzumab ozogamicin; NK = natural killer; R/R = relapsed or refractory.

## Personalized medicine tools and translation in applied hematology

During the past 2 decades, a number of tools and approaches related to personalized medicine have been developed and have been translated into clinical practice in patients with hematologic neoplasms. Examples are shown in Table 5 and include whole-genome sequencing and molecular screens for mutation profiles predicting prognosis, responses to therapies, and/or the risk to develop side effects in individual patients. Other examples are antibody-based cell phenotyping to predict responses of neoplastic cells to therapy (eg, antibody-based or cell-based therapies) in individual patients, or multidrug testing approaches predicting responses of patient-derived cells to drugs or drug combinations, and a comparison between normal and neoplastic cells (therapeutic window).^72-79^ Such studies may predict responses of neoplastic cells to certain drugs or drug combinations in individual patients. Pharmacological studies can also confirm that the patient is adherent and that drug intake leads to an effective trough level (to avoid under- or over-treatment).

**Table 5:** Tools and Approaches Employed in Personalized Medicine in Blood Cell Neoplasms.

**Diagnostic/prognostic (examples**)
• Whole-genome sequencing screens for mutation profiles/patterns to predict prognosis, responses to therapies, and/or the risk to develop side effects in individual patients
• Specific genetic screens for mutation patterns to predict prognosis, responses to therapies, and/or the risk to develop side effects in individual patients
• Antibody-based cell phenotyping to predict responses of neoplastic cells to therapy (eg, immunological antibody-based or cell-based therapies) in individual patients
• Multidrug testing approaches predicting responses of patient-derived cells to drugs or drug combinations: comparing normal and neoplastic cells (therapeutic window)
• Monitoring of MRD in individual patients (using patient-specific and/or disease-related MRD markers) before and during therapy
• Pharmacological studies to predict responses of neoplastic cells in individual patients or to confirm an effective trough level-range or to reveal under- or over-treatment
• Multiparametric scoring systems predicting overall survival, progression-free survival, or both, in patients with hematologic neoplasms
**Therapeutic (examples**)
• Application of specific targeted drugs or drug combinations (combination or sequential) based on the unique composition of drug targets identified in neoplastic cells in individual patients
• Dose and time adjustments of therapies based on individual, patient-specific variables such as age (eg, age-adjusted dose reductions), comorbidities, previous therapies, organ (eg, bone marrow) function, ethnic background, social status, or availability of medical facilities
• Administration of drug combinations based on preclinical and clinical data to promote antineoplastic effects of individual drugs and to minimize side effects of individual drugs
• Computer-based drug-drug interaction assessments and resulting recommendations to apply (or not to apply) certain drugs in combination (eg, to avoid side effects)

MRD = minimal residual disease.

One of the best examples demonstrating the applicability and impact of precision and personalized medicine tools is CML. This disease is characterized by multiple mechanisms of stem cell resistance and profound genetic instability. As a result, the CML stem cell genome exhibits extensive plasticity and the mutation status can “adjust” to new therapies based on expansion of subclones expressing resistant mutants of *BCR-ABL1* and other resistance mechanisms. Clinically, this was soon appreciated by the detection of point mutations within *BCR-ABL1* during treatment with BCR-ABL1 inhibitors. And whereas many of the mutant forms of BCR-ABL1 are responsive to second and third generation BCR-ABL1 TKI, even more mutations (presumably present in preformed subclones prior to therapy) may be detected during therapy, including *BCR-ABL1* compound mutations (1 or more secondary *BCR-ABL1* mutations in the same *BCR-ABL1* alleles). Moreover, it turned out that the new TKI also exert a number of clinically relevant side effects. However, these side effects were not observed in all patients. With time and experience, the community appreciated which TKI-induced adverse events are associated with which risk profiles. As a result, TKI are now applied with great precision in CML therapy and can be used sequentially or even in rotation to balance between the risk of side effects and optimal effects on all relevant subclones, following the principles of personalized medicine.^21–26^

Apart from CML, there are several other examples in applied hematology where both high-end precision medicine tools and personalized medicine strategies have successfully been translated into clinical practice. In fact, for many of the clinically important and disease-specific mutant forms of oncogenic kinases, effective kinase inhibitors have been developed in recent years. In many disease models, these kinase blockers have been successfully translated into clinical practice.

Multiparametric scoring systems that can assist in prognostication are a major tool in personalized medicine. In fact, in almost all hematologic neoplasms, 1 or more multiparametric scoring systems have been developed to better predict survival and/or other outcomes in the past 30 years. In many instances, multiple scores can be applied, and each of these scores is optimized (or can be optimized) to predict overall survival, progression-free survival, or the chance to benefit from certain therapies, such as hematopoietic stem cell transplantation. Depending on endpoints, these scores include certain variables. For example, a score that should predict overall survival or survival after intensive therapy usually includes age. Some of the scores have been designed to predict the patient’s risk of developing serious adverse events during therapy. This area of personalized medicine is of utmost importance when patients qualify for intensive therapies, such as stem cell transplantation, and may be at (high) risk of developing serious adverse events or even die from such intensive approach. Similar scores may also be helpful in predicting the risks and chances of patients treated with novel toxic chemotherapies, antibody-based therapies, or cell therapies.

It is also worth noting that several of the established scores are already supported by computer programs, and we are convinced that personalized medicine approaches will soon refine (or complement) these scores, thereby assisting the hematologist in daily practice. With regard to individualized treatment, machine learning/robot-supported algorithms are also expected to aid us in applied hematology and to produce diagnostic estimates, preliminary diagnoses, and even recommendations for treatment. However, such machine-based learning processes require larger numbers of patients. Moreover, because of the many different patient-related factors, disease-related features, pharmacologic variables, and drug-drug interactions, and because all these factors may change quickly with time, it will be extremely difficult to replace the physician (hematologist) by exclusively machine-based recommendations and algorithms. Similarly, it may be difficult or impossible to develop a computer-based program that can rapidly establish the correct diagnosis, the optimal management plan, and the optimal therapeutic intervention in polymorbid patients who have unclear symptoms and take multiple medications, even when employing optimized virtual medical coach-programs. In many instances, only the experienced physician who knows the patient and all relevant cofactors (often for many years) will be able to ask the right (relevant) questions, to appropriately feed the supporting machines, draw the right conclusions from the information provided, ask for the appropriate diagnostics, and arrive at the correct diagnosis.

A special field in translational hematology is the development of novel and better molecular markers (biomarkers) that either have diagnostic potential or are of prognostic significance. Whereas precision medicine tools and biobanking systems support the development of biomarkers and their validation, only clinical application in studies and finally in daily practice, will guide us in the optimal application and will reveal the real value of these biomarkers in personalized hematology. A summary of most relevant personalized medicine tools is shown in Table 5.

An important question is when and in which patients should personalized medicine be preferentially applied in clinical practice. Whereas this question is difficult to answer in general, more and more data and observations suggest that precision medicine and personalized medicine in hematologic neoplasms can, in principle, be applied in all patients and in every type of neoplasm. In fact, even in indolent neoplasms, such as the chronic stable leukemias, low-risk myelodysplastic syndromes, or chronic and stable myeloproliferative neoplasms, it makes sense to apply prognostication-related tools, to estimate the actual (and individual) risk, and to introduce prevention or early therapy, with the aim to avoid progression and secondary complications, to extend survival, and/or to keep the quality of life as high as possible.

In the past 15 years, we and others have characterized potential prephases of hematologic (myeloid) neoplasms defined by molecular (somatic) lesions with or without certain blood count abnormalities or other signs of a clonal process.^80-83^ One good example is clonal hematopoiesis of indeterminate potential, also known as age-related clonal hematopoiesis.^80-83^ Another example is monoclonal B-cell lymphocytosis, a neoplastic condition that usually shows a stable course but may progress or may be complicated by infections. In all these conditions, precision medicine tools and personalized medicine approaches should be considered and may lead to improved diagnostic and prophylactic approaches. In this regard, it is worth noting that in the future, precision medicine and personalized medicine tools should not only be offered in specialized university centers but also in peripheral centers and hospitals.

## Remaining questions and proposed strategies to solve some of the issues in precision medicine and personalized medicine in hematology

Despite rapid developments in the field of applied hematology, a number of questions remain concerning the application and value of precision medicine tools and personalized medicine strategies in daily practice.^1-7^ One obvious question is whether all tools and technologies that have been developed in recent years to roll out precision and personalized medicine in hematology in various countries will be affordable for our healthcare systems. For example, it remains unknown how the rapidly increasing numbers of markers, targets, drugs, and technologies can be implemented in practice and can be maintained (covered) by our healthcare systems. A good example is the CAR-T cell technology that is effective but extremely complex, costly, and dependent on a high-level cell therapy unit on site. Other examples are various gene therapies, transplantation technologies, or complex surgical approaches.

There is also a rapidly increasing number of complex clinical trials that are increasingly cost-intensive and are also accompanied by an equally expensive and constantly growing number of regulatory and legal requirements. The question is how all these trials and studies in more and more (precisely defined) patient subsets can be carried out and are afforded by companies, participating centers, and our healthcare systems. To complicate matters, personalized medicine is now looking (must now look) into smaller subsets of patients (defined by patient-related and/or disease-specific features) in special trials and drug-testing programs to address the specific medical need. Here, the only solution may be to reduce the overwhelming bureaucracies and (sometimes unnecessary) regulations that are intended to protect patient safety but, in reality, may hurt patients as they hamper progress. This is particularly true for small-sized clinical trials and the implementation of complex technologies used in daily routine practice. Our faculty is of the opinion that the conduct of small interventional pilot trials (up to 15 patients managed/treated in the same way, eg, with the same therapy) with reduced bureaucracy would not only reduce costs but would also promote precision/personalized medicine by rapidly testing novel promising strategies in various patient subsets even in rare neoplasms (Table 6). And in the case of encouraging results obtained in such pilot trials, the concept could then be tested in larger multicenter studies or even global trials.

**Table 6:** Strategies to Limit the Costs in Personalized Medicine and Precision Medicine in Hematology.

• Global data exchange and multicenter or global validation of new tools and techniques
• Employing larger preexisting databases to test and validate new tools and technologies
• Establishing well-organized national, international, or even global competence networks
• Establishing reference centers for technologies and special topics in precision medicine and personalized medicine
• Establishing centralized first-class diagnostic laboratories supported by robot- and computer-assisted high-capacity testing facilities (national/regional level)
• Establishing local core facilities for major tools and technologies in each center and region
• Establishing the concept of small (academic and company-driven) pilot trials with minimal regulatory, administrative, bureaucratic, and financial investment
• Implementing uniform and evidence-based precision medicine and personalized medicine approaches in local centers through CCCs and tumor boards
• Establishing multicenter national or international (global) real-life data registries
• Employing global databases to implement personalized medicine approaches for optimal drug application and patient protection (eg, databases describing drug-drug interactions)
• Establishing robust biobanking systems in major hematology centers and organizing multicenter biobank systems through which sample series can be analyzed even in rare neoplasms
• Optimal information of patients and education of doctors to minimize unnecessary referrals, tests, and therapies and to diagnose and treat early and thus most effectively
• Telemedicine approaches which can support diagnosis and prognostication and thereby spares (additional) visits and referrals
• General pricing models that define a maximal (precalculated and thus predefined) price per approach, test, or drug in each region and country

CCC = comprehensive cancer center.

There are also other possible ways to reduce costs locally or globally in the fields of precision medicine and personalized medicine.^84,85^ One proposed approach is to implement a general pricing model where the maximal price of each drug is precalculated and thus predefined.^84^ A summary of these strategies aimed at reducing costs is shown in Table 6. One strategy is to rapidly exchange data and knowledge among centers and universities or even at a global level. In fact, global data exchange and multicenter or global validation of tools and techniques would most probably save local budgets through concerted predefined testing (Table 6). Another related strategy is to support the establishment of well-organized national and international competence networks (eg, US-wide and/or European-wide collaborative networks) for each disease, including rare diseases, where experts provide all available tools and facilities relevant to personalized medicine and precision medicine. Typically, such networks include highly specialized reference centers as well as centers of excellence (Table 6). Some of these centers will serve as major referral centers for challenging cases requiring complex care and special tools (reference center). Depending on the prevalence of the disease or condition, 1 or 2 such center(s) per country or region should be sufficient to cover the topic with appropriate resources and tools. It is important that such sites are equipped with high-end facilities. For example, primary referral laboratories (reference center status) should be first-class diagnostic centers supported by (robot + computer-assisted) high-capacity testing facilities and related technologies. In addition, all active centers working in the fields of precision medicine and/or personalized medicine should establish local core facilities for major tools and technologies. In larger centers and universities or university hospitals, it is equally important to implement evidence-based precision medicine and personalized medicine approaches and tools in collaboration with local comprehensive cancer centers and to implement precision medicine and personalized medicine strategies via local tumor boards or similar decision panels (Table 6). In the future, such precision medicine tools and strategies should then also be rolled out on a broader basis in the healthcare systems (eg, in peripheral hospitals) if possible.

Another important tool in precision medicine is registry data networking. In fact, establishing multicenter national or international (global) real-life data registries for various groups of patients may help saving costs by learning from real-life–based data sets. This may be particularly important for the management of rare diseases.

Another potential cost-saving strategy in precision and personalized medicine may be to implement computer-assisted tools and telemedicine approaches through which local visits might be reduced and the patient and doctor could still communicate with almost the same quality of assessment and care. This new way of moving forward in applied medicine has been pushed forward by the current severe acute respiratory syndrome coronavirus 2 crisis.

Finally, open innovation in science strategies involving patients and relatives may sometimes help. In fact, involving the patients’ opinions and patient self-support groups in the development of precision medicine and personalized medicine projects may reveal additional aspects and unmet needs, and may lead to an extra gain of knowledge for both the patients and their care providers.^86,87^ In this regard, it is important to provide wide access to high-quality, affordable, precision medicine tools and approaches through major hematology organizations like American Society for Hematology or European Hematology Association (EHA).^86-89^

## Concluding remarks and outlook

The terms “precision medicine” and “personalized medicine” are indicative of an emerging revolution in our scientific communities and healthcare systems. In fact, our improved knowledge about disease-specific variables, patterns, and patient-related factors are now increasingly used to ensure optimal diagnosis, prognostication, and therapy in most or all patient subsets in an “individualized” manner in various neoplasms. Hematology is an excellent example highlighting the possibility to employ multiple disease- and patient-related parameters to develop improved applications and management in defined patient cohorts. Thus, personalized medicine has engendered a platform to build innovative approaches based on our improved knowledge of disease causality, progression, and treatment resilience. In the current article, we provide definitions for the terms “precision medicine” and “personalized medicine” in hematology, based on an in-depth discussion of their meaning and use in daily practice. Common use of these terms should facilitate communication in research, applied medicine, and in the public, and should thereby support the scientific development in the field. In addition, we propose strategies for a broader and more rapid implementation of precision and personalized medicine tools in daily practice. Although the suggestions and strategies proposed by us and by others are also aimed at reducing costs, a main question for the future will be whether all precision and personalized medicine tools and approaches will be affordable for our healthcare systems.

## Sources of funding

This project was supported by the Austrian Science Fund (FWF)—grants F4701-B20 and F4704-B20. UJ was supported by grant LS16-034 from the Vienna Science and Technology Fund (WWTF).

## Disclosures

The authors have no conflicts of interest to disclose.
